# Supplementary material for: Development of a Novel Anti-CD44 Variant 7/8 Monoclonal Antibody, C44Mab-34, for Multiple Applications against Oral Carcinomas
Source: Biomedicines. 2023 Apr 5;11(4):1099. doi: 10.3390/biomedicines11041099 (PMC10136282; doi:10.3390/biomedicines11041099)
Supplement: Supplementary file 1 [file biomedicines-11-01099-s001.zip › rev supple Fig. S1 and S2.pdf]

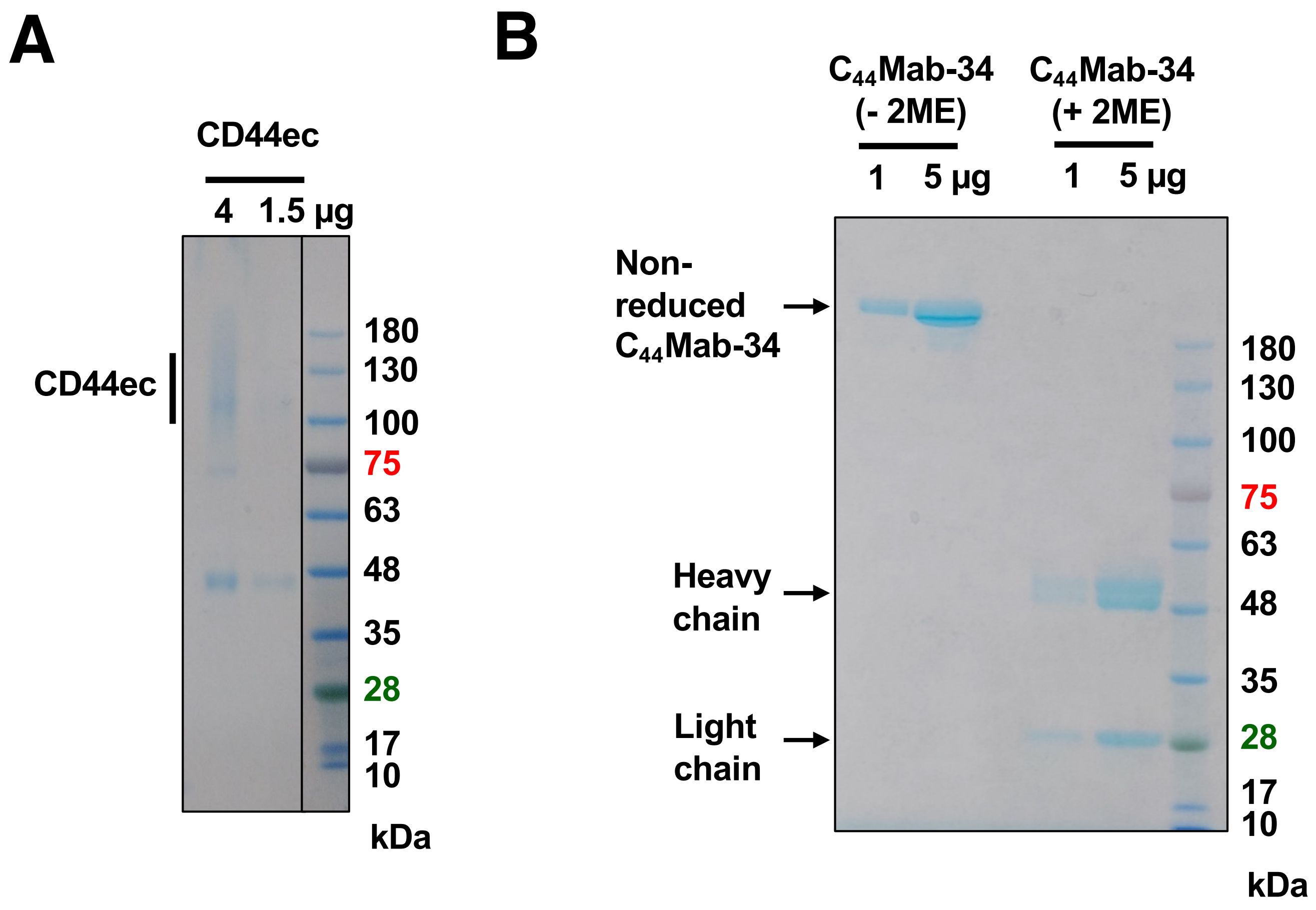

Supplementary Figure S1. CBB staining of CD44ec and C_{44}Mab-34.
(**A**) The purified CD44v3–10 ectodomain (CD44ec, 4 and 1.5 μg) was denatured by sodium dodecyl sulfate (SDS) sample buffer (Nacalai Tesque, Inc.) in the presence of 2-mercaptoethanol (2-ME). Proteins were separated on 5%–20% polyacrylamide gel (FUJIFILM Wako). The gel was stained by Bio-Safe CBB G-250 Stain (Bio-Rad Laboratories, Inc.).
Note: CD44ec is broadly detected due to its glucosylation.
(**B**) C_{44}Mab-34 (1 and 5 μg) were denatured by SDS sample buffer in the presence or absence of 2-ME. Proteins were separated on 5%–20% polyacrylamide gel. The gel was stained by Bio-Safe CBB G-250 Stain.

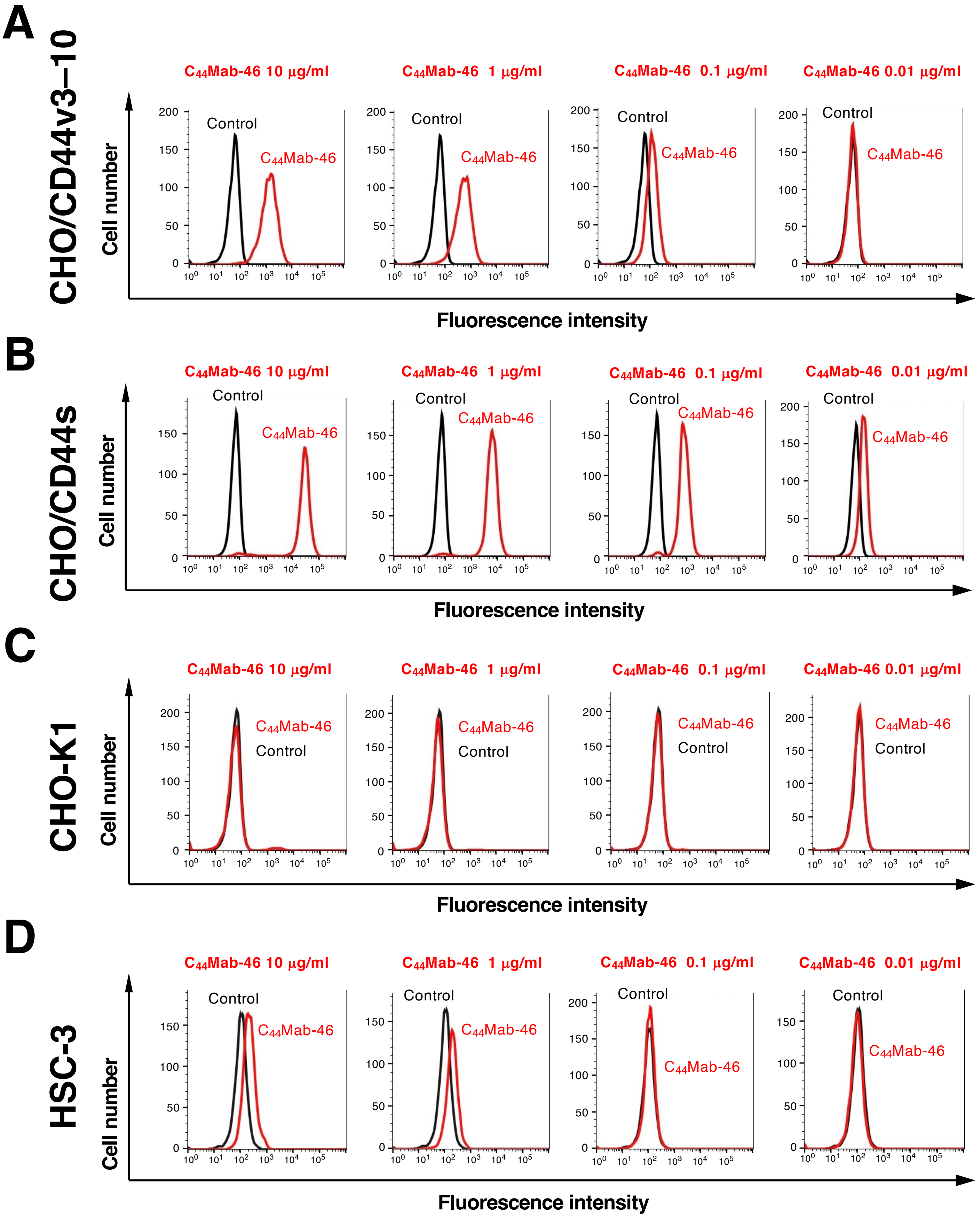

Supplementary Figure S2 Recognition of CHO/CD44s, CHO/CD44v3–10, and HSC-3 by C_{44}Mab-46 by flow cytometry. CHO/CD44v3-10 (A), CHO/CD44s (B), CHO-K1 (C), and HSC-3 (D) were treated with 0.01-10 µg/mL of C_{44}Mab-46, followed by treatment with Alexa Fluor 488-conjugated anti-mouse IgG (Red line). The black line represents the negative control (blocking buffer).
